# Adapting the rhizome concept to an extended definition of viral quasispecies and the implications for molecular evolution

**DOI:** 10.1038/s41598-024-68760-6

**Published:** 2024-08-02

**Authors:** Carlos Raico Landa, Ascensión Ariza-Mateos, Carlos Briones, Celia Perales, Astrid Wagner, Esteban Domingo, Jordi Gómez

**Affiliations:** 1 Laboratory of RNA Archaeology, Instituto de Parasitología y Biomedicina “López-Neyra” (CSIC), Avd. Conocimiento 17, 18016 Armilla, Granada Spain; 2 Centro de Biología Molecular “Severo Ochoa” (CSIC-UAM), Campus de Cantoblanco, Madrid, Spain; 3 Department of Molecular Evolution, Centro de Astrobiología (CSIC-INTA), Madrid, Spain; 4 Centro Nacional de Biotecnología (CNB-CSIC), Campus de Cantoblanco, Madrid, Spain; 5 Department of Clinical Microbiology, IIS-Fundación Jiménez Díaz, UAM, Madrid, Spain; 6 Instituto de Filosofía del CSIC, Madrid, Spain

**Keywords:** Viral evolution, Molecular evolution

## Abstract

The rhizome concept proposed by Gilles Deleuze and Félix Guattari offers a novel perspective on the organization and interdependence of complex constellations of heterogeneous entities, their mapping and their ruptures. The emphasis of the present study is placed on the dynamics of contacts and communication among such entities that arise from experimentation, without any favored hierarchy or origin. When applied to biological evolution, the rhizome concept integrates all types of heterogeneity resulting from “symbiotic” relationships among living beings (or their genomic material), horizontal genetic transfer, recombination and mutation, and breaks away from the approach that gives rise to the phylogenetic tree of life. It has already been applied to describe the dynamics and evolution of RNA viruses. Thus, here we introduce a novel framework for the interpretation the viral quasispecies concept, which explains the evolution of RNA virus populations as the result of dynamic interconnections and multifaceted interdependence between highly heterogeneous viral sequences and its inherently heterogeneous host cells. The rhizome network perspective underlines even further the medical implications of the broad mutant spectra of viruses that are in constant flow, given the multiple pathways they have available for fitness loss and gain.

## Introduction

There is a growing acknowledgment that organisms are complex societies or ecosystems comprising different species (and, in particular, including their microbiotas), as expressed by Scott G (2019) "We complete each other"^1–3^. For instance, cows and termites lack the genes needed to digest cellulose or lignin respectively, therefore these essential functions are provided by their cohabiting bacteria^1^. Moreover, experimental studies indicate that in these cases cohabitants not only share functions but also influence animal development^4^. Much like a Russian doll, even simple entities (such as a single cell eukaryotic organism) exhibit a mosaic-like composition, with mitochondria and chloroplasts and other organelles resulting from the integration of ancient freely living organisms^5^. The eukaryotic nucleus has been recently proposed to have originated from a fusion event between archaebacteria and a giant virus^6^. At the molecular level, heterogeneity is present in a wide range of agents that maintain a relationship of molecular parasitism, cooperation and conflict^7–10^. For instance, various genetic elements, such as retrotransposons and maternally transmitted cytoplasmic genes (i.e., mitochondrial), do not adhere to the same rules of hereditary transmission as cellular genes^11^ (genetic conflict). The agent for hepatitis delta only infects the human liver if co-infected with the hepatitis B virus^12^, and the Sputnik virophage behaves as a parasite of the mimivirus, a protist giant virus^7^. Also, after a virus infection, the reactivation of ancient molecular activities or configurations of the cell that are typically not expressed in uninfected cells may represent another type of relation within the cell^13^.

In an evolutionary context, rather than solely focusing on individual competition for resources, there is a growing emphasis on the intricate networks of cooperation and interpenetration that occur both between and within organisms, lineages, and assemblages^2^.These entities engage in continuous biological communication. Coordination between assemblages of organisms from different lineages relies on shared signal-mediated interaction rules^14^ which are possible because there exist a previous historical framework of coexistence of living beings^15^. Some of these rules, such as the genetic code, which operates mostly like a syntax-dependent language, are nearly universal. However, most other rules may differ significantly between distinct agents^16^ and context, for example the rules that govern where and when the ribosome should initiate protein translation of a messenger RNA (mRNA)^17^ . These other signal languages are highly context- and consortia-dependent, formally resembling everyday human language^14^. The historically grown communicative practices of molecular communities are a prerequisite for the organization and coordination of different molecular agent/consortia, and RNA viruses have been described to be a part of these^18,19^.

In contrast to the hierarchical representation of phylogenetic relationships based on single genes (the classical example being ribosomal genes^20^) the evolutionary connections between the genomes of such organisms and their associated symbionts cannot be depicted as a bifurcating tree^21,22^. Moreover, the absence of a defined biological individuality, whether anatomical, physiological, genetic, evolutionary or immunological, is a new paradigm in biology^2^.

**Figure 1:**
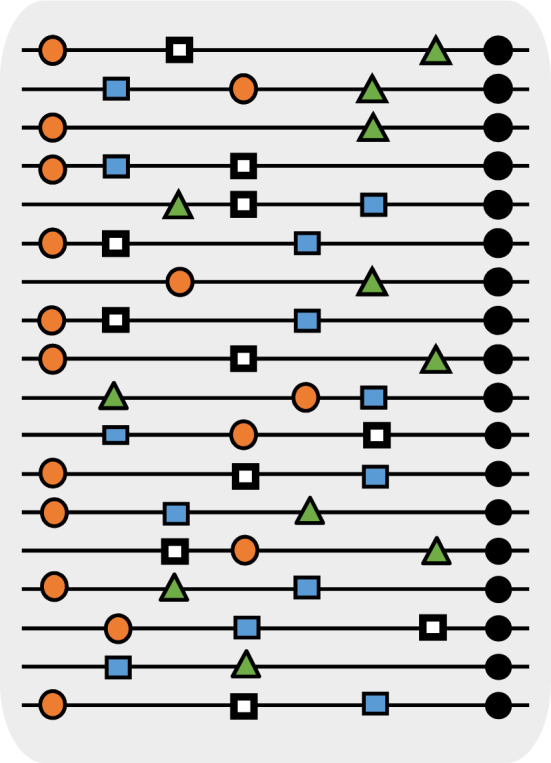
Scheme of viral quasispecies. Each line represents a viral genome, and the symbols on the lines show point mutations. The set corresponds to the population of a RNA virus isolated from a single patient or cell culture.

RNA viruses (both ribo- and retroviruses) have a property that distinguishes them from DNA viruses and cells: their population structure is decisively influenced by a significantly high error rate (of about 1 mutation introduced per 10,000 nucleotides copied from a template genome). Thus, any organism infected by an RNA virus contains a very high number of copies of similar, but not identical, nucleotide sequences called “mutant spectra”, “mutant swarms”, or “mutant clouds”. Each individual genome from the cloud, with its distinct nucleotide sequence, has a transient existence. In virology, the concept of quasispecies has been adopted to refer to complex distributions of closely related genome variants subjected to genetic variation, competition, and selection, and which act as a unit of selection^23^ (Fig. 1).

The recognition of quasispecies dynamics revealed several implications for RNA viruses. They can be divided in: (i) those that redefined the wild type virus as a set of genomes, versus the previous view of being an individual genome, (ii) those that evidenced molecular mechanisms of adaptability to new environments, and (iii) those that promoted new means of antiviral interventions, such as the requirement of combination therapies to minimize selection of escape mutants, or the use of lethal mutagenesis (virus extinction by excess of mutations, in confirmation of a tenet of quasispecies theory) [review in^24^]. Of these different facets, in the present study we will focus on those that most contribute to the participation of viral quasispecies in the rhizomatic network of life.

Gilles Deleuze and Félix Guattari (referred to as D&G hereafter)^25^ coined the term “rhizomatic” for their epistemological approach in the book “A Thousand Plateaus” (1976)^25^ , in which they put forward a series of principles in order to theorize about phenomena with changing, heterogeneous, nonhierarchical, and decentralized characteristics. Their approach is framed within a classic debate in the history of Western philosophy, namely the discussion regarding the prevalence of stability over change, or vice versa, as the ultimate foundation of reality, i.e. of everything that exists (see box 1).

We will review the reception of this concept in biology and then examine how it fits into the definition of RNA virus quasispecies.

## The rhizome concept

The rhizome can be perceived as a descriptor of reality as a process in which the fundamental aspect is its dynamic, ever-changing nature. Based on this conception, “neither the structures of such processes nor their completed products merit the same ontological status as the processes themselves”^26^.

**Figure 2:**
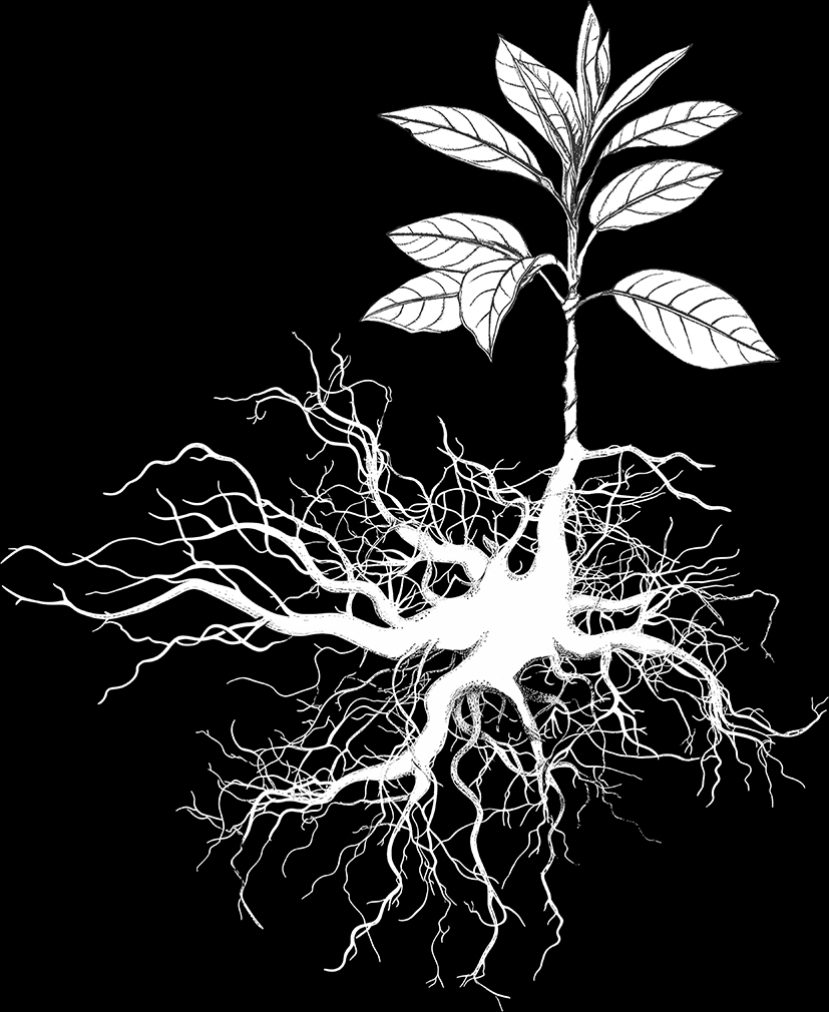
Artistic recreation of the rhizomatic quasispecies chimera. The illustration depicts the sequence space occupied by a viral quasispecies, where sequences are not organized like a branching tree but rather as numerous rhizomatic extensions akin to that of ginger or potato terrestrial plant parts.

D&G illustrate their conception of reality in analogy with rhizomatous stems of certain plants (Fig. 2), such as those of the grass or the potato, since, in them, “what appears” (the rhizome shoots) is the result of the multiple, horizontal, and hidden interconnections that constitute their “rhizomatic background” (ontological background). The concept of rhizome serves as a descriptive model that captures the process and creativity that define the living world. It is embedded in a process ontology where the classical concept of substance, which assumes individual and stable entities as the basic components of reality is replaced by the notion of multiplicity, which conceives the process-based entities themselves as dynamic relational structures^27^ The concept of the rhizome challenges traditional organizational structures, our logic, and grammar. Thus, there is no universal grammar, as each language grows differently. The rhizome offers multiple points of entry to reality, resembling a decentered and interconnected web of relations, which contrasts with the hierarchical model of a tree^28^, and specifically a Porphyrian tree^15^.

It is the potential difference between the interacting entities or structures, and its consequent resolution (actualization, updating, integration), that generates the dynamism of reality as a process. Moreover, multiplicity is not contemplated as merely a conglomerate or collection of discrete and isolated parts, but rather as a plurality of potentialities susceptible to be actualized due to the heterogeneity of possible connections between different entities. This process involves an ongoing dialectical progression, as described by D&G, between what they refer to as the “extensive-actual” (what currently exists) and the “intensive-virtual” (what holds the potential based on the interacting forces). In some respects, the concepts of “intensive-virtual” and “extensive-actual” resemble the Aristotelian notions of “exist in potency” and “exist in act”. However, the key difference between the Deleuzian and Aristotelian theories lies in the fact that, whereas Aristotle proposed that potentialities are inherent to different substances, D&G consider them to be formed by the various connections and relationships established during the process^29^. We illustrate this point with an example in box 2.

Reality can thus be understood as being rhizomatic because it consists of a *virtually potential interaction* between “everything”. This interaction differs from the stable and unquestionable (vertical) hierarchies, decentralizing the system and promoting a horizontal/rhizomatic relationship, which gives rise to the constant generation of inexorably interdependent multiplicities. The genesis of reality is framed, shaped, and expressed in the dynamic process that has taken place when resolving the potential difference that gives rise to the passage from the intensive-virtual to the extensive-actual, an example of which is provided in box 3.

## The rhizome concept applied to modern biology

Several authors have applied the rhizome concept to biological evolution. Didier Raoult, for example, highlighted two key discoveries which indicate that the rhizome concept is a better descriptor in terms of biological evolution than the diversification by branching typical of phylogenetic trees: horizontal gene transfer^30^ (HGT), and orphan genes (ORFans). With respect to HGT, while this type of transfer mechanism was initially observed only in bacteria, where the concept has found numerous examples, it was soon identified in the three domains of life, frequently involving viruses as carriers for the transferred genes^31^. As an example, it has been proposed that “the human genome is a mosaic of genes with eukaryotic, bacterial, […] and viral origin”^31^. Other factors, such as exon exchange in unicellular organisms (known as exon shuffling), also contribute to the broad idea of genetic mosaicism^32^. Moreover, large-scale genomic testing has revealed that many genes could have arisen through fusion, degradation, or other events, and that some of them are found in only one organism^31^. These new genes were dubbed orphan genes as they do not have any homologs outside their given species or lineage^33^.

These findings were initially presented as anomalies that had to be assimilated into existing theories of evolution^30,31,34,35^. The traditional representation of diversification of species as a phylogenetic tree is based on a hierarchy that portrays the outcome of evolution as successive divisions branching out from a common origin or ancestor. It also depicts the stability of each branch, as they diverge from one another, and the trend is to separate further over time. However, given the increasing evidence for HGT, this linear, phylogenetic representation should be complemented by the complex rhizome concept^31,34–37^.

Similarly, Eugene Koonin ^38^ proposed that some of the major evolutionary biological transitions address the alternation between two stages of evolution. The first of them is a stage of rapid evolution, which is characterized by the swift and disproportionate exchange and recombination of genetic material, resembling a rhizomatic structure and serving as the foundation for various pivotal evolutionary transitions: the emergence of complex RNA and protein folds, the diversification of viral types, the origin of archaea and bacteria from LUCA, the branching of key lineages within these prokaryotic domains, the development of eukaryotic supergroups, and the classification of various animal phyla. Subsequently, in each of these cases, such a rapid phase gives way to a second, evolutionarily slower and more tree-like (arborescent) evolutionary pattern. This dual model combines the concepts of rhizomatic and arborescent evolution into a unified theory.

## Principles of the rhizome

The rhizome principles are guiding principles intended to shape the understanding of dynamic and ongoing phenomena. In essence, these principles offer a framework for theorizing changes and processes^29^.

**1. and 2. Principles of connection and heterogeneity:** these two basic rules state that “any point of a rhizome can be connected to any other, and must be”^25^.

Despite its metaphysical undertones, these principles oppose any form of hierarchical organization; it is inherently anti-hierarchical. They emphasize that the rhizomatic model permits unrestricted connections between points, thereby creating a dynamic network in which any point can interact with any other, thus the rizhome constitutes a decentralized whole that constantly reorganizes itself according to the context.

We understand that the order of these principles (1 and 2) is such because both of them are logically interdependent and based on what was explained above: more connections entail more heterogeneity, and more heterogeneity implies the potential for new connections. If the concept of substance is replaced by that of multiplicity, and the resulting entities and structures are interconnected and interdependent, then all forces and matter influence all other forces and matter via multiple connections. Thus, the influence and interdependence inherent to the process are also the basis for its dynamism and creativity (innovation). Furthermore, the connections in a rhizomatic paradigm become heterogeneous and generate heterogeneities (products, functions, and structures) with heterogeneous meanings^25^ and which are constantly being spawned in successive stages of the process.

**3. Principle of multiplicity:** this tenet claims that “it is only when the multiple is effectively treated as a substantive, ’multiplicity’, that it ceases to have any relation to the One as subject or object”^25^.

This principle reiterates the interdependent character of the entities and structures resulting from the process, and the virtual nature deduced from this interdependence. Let us take a human being as an example. We use the term “human” to refer to an entity that is apparently characterized as being one human, subject or object. However, there are good reasons to conceive the human entity as a structure derived from interdependent processes. A human being, as an overall process, rely on the symbiotic relationship between the body (composed of cells of the species *Homo sapiens*) and the intestinal, skin and mucosal microbiota (belonging to very different sets of bacterial/archaeal/eukaryotic species, as well as viruses) in order to complete various metabolic processes^39^. Additionally, the human is also influenced by his/her relationship with domestic animals and their products, such as fermented milk for example^40^. This connection not only links humans with animals but also with their bacteria, hormones, enzymes, food, and, collectively, with material culture (production of cheese, wool, stables…).

From this perspective, the concept of “human being” alludes to a multiplicity of processes that maintain human homeostasis as a result of their mutual connection and interdependence (reality understood as a multiplicity).

**4. Principle of asignifying rupture:** “against the oversignifying breaks separating structures or cutting across a single structure”, this principle proposes that a “rhizome may be broken, shattered at a given spot, but it will start up again on one of its old lines, or on new lines”^25^.

While the traditional, hierarchical idea of reality depends on certain principles established as axioms upon which everything else is founded, the rhizome concept, with its decentralized system, offers the possibility of reorganization, subject to the continuous development of the process. This is because the functions are determined by the relationships established between the elements that make up the process rather than being derived from a founding instance or particularity. As such, certain elements may be self-sufficient with respect to others, provided that the relationship constructed between them is capable of maintaining the function of the whole. So, the principle of an asignifying rupture highlights that the relationship between elements determines and dynamizes the process’ functions. An example of asignifying rupture can be found in a colony of ants: even if most of the population is destroyed, reconstruction and renewal never cease^25^.

**5. and 6. Principles of cartography and decalcomania:** these rules assert that “a rhizome is not amenable to any structural or generative model”^25^.

In relation to the rhizome representation, D&G methodologically compared a “map” (representing the model of a rhizomatic conception of reality) to a “tracing” (from the classic conception of reality) and suggested that tracing hierarchizes, organizes, stabilizes, and neutralizes the multiplicities according to their own lines of meaning^25^. This epistemological stance can be illustrated by using the example of a traditional enterprise based around a clear hierarchical order and well-defined roles of its employees. Each of them has a specific place and role within the hierarchy, while the decisions and information flow downwards from the top by means of the predefined organizational structure. Such an enterprise follows a “logic of tracing”, where a given structure and certain procedures reproduce themselves as they follow a predetermined template. Therefoer, in this traditional model, the supervisor/operator 1, operator 2, etc. relationship is a tracing of the chief executive officer (CEO)/ chief operating officer relationship, chief financial officer, etc. Tracing therefore presupposes itself and is reproduced in its activity.

In contrast, “what distinguishes the map from the tracing is that it is entirely oriented toward an experimentation in contact with the real.”^21^: the map is construction, rather than reproduction. Unlike tracing, which involves reproducing existing knowledge or skills, in this context mapping means actively constructing new knowledge by way of experimentation with the real world. It is about doing, not just knowing, and it demands a hands-on, active approach. D&G argue that mapping requires an active capacity (*performance*), while tracing requires a previously learned skill (*competence*)^25^.

The internet can be used as a current example of a map as it consists of websites, social media, communication protocols, and information connected via a network of multiple and redundant, channels but with no hierarchical or central structure. There is not a single path to follow while navigating the internet, and the information branches off and interconnects in various directions via hyperlinks. As such, the internet forms a map insofar as it possesses open-ended possibilities in terms of navigation, connection, and reorganization. This structure also provides more robustness the network.

“The map is open and connectable in all of its dimensions; it is detachable, reversible, susceptible to constant modification”^25^. Given that it assumes an active capacity, the map’s elements are open to reinterpretation and reorganization. A tracing, on the other hand, as a competence, assumes certain prior competences as part of its reproduction: the tracing must be known in order to do the tracing. However, the fact that the ontological background is rhizomatic is not incompatible with the emergence of tree-like structures within the system, as previously discussed. Indeed, traces can be temporarily compatible with maps as they are always susceptible to alteration and restructuring, as emphasized by D&G^25^.

## Principles of the rhizome with respect to viral quasispecies

We will now assess whether the principles of the rhizome are applicable to experimental findings in viral quasispecies (box 4), with the aim of exploring if what has been termed quasispecies dynamics can be interpreted from a process-based perspective.

### In terms of 1. and 2. Principles of connection and heterogeneity

The first experimental study that defined the structure of quasispecies in RNA viruses^41^ did so by means of a direct method (involving RNA fingerprinting^42^) for detecting mutations of individual clones isolated from a population of *E. coli* Qβ phages, as well as of the whole population. This study concluded that the population contained a heterogeneous mixture of sequences with an average of one or two mutations among them^41^ thus implying that the genome of the Qβ bacteriophage could not be described as a single, defined structure, but rather as a set of genetic variants present in the population at different relative frequencies. This way of structuring the genetic information accommodated the definition of quasispecies conceived for the theoretical replicons thought to have participated in the origin of life^43^. Besides RNA bacteriophages, the animal and plant RNA viruses studied to date have also been shown to replicate and evolve according to quasispecies dynamics^44^. Such population heterogeneity can even be observed among the viral sequences replicating in a single cell^45^.

**Figure 3:**
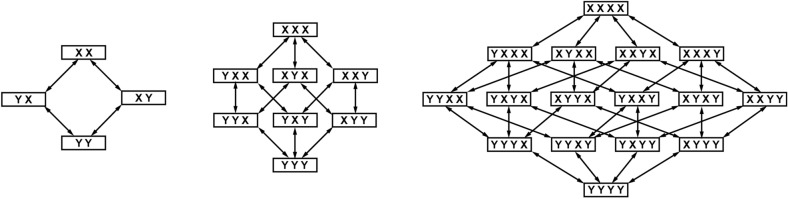
Construction of a sequence space using two digits, X and Y. Each sequence is positioned at a point in space, at a distance from other sequences given by the difference between the digits (i.e., number of point mutations separating both sequences). For example, a difference of 1 mutation results in a separation of 1 cm, while 2 mutations lead to a 2 cm separation, and so on. As the sequence length increases, a shape known as a hypercube is formed. ^46^ Modified from

Ordering the genetic variants of a viral quasispecies spatially in a hypercube helps to illustrate the connectivity of viral sequences (Fig. 3). The hypercube’s multidimensional structure shows that all the vertices (each of them corresponding to a given viral sequence) may be connected to each other via (more or less) mutations that occur during the error-prone RNA replication^46^. The high mutation rate typical of RNA viruses implies that each time a sequence multiplies it immediately connects with its nearest neighbors (at a genetic distance of 1) and, although less often, it can also connect with more distant nodes in the sequence space via copies that contain multiple mutations^46^. Figure 2 shows an artistic representation of the distribution of variant sequences of viral quasispecies in a rhizomatic n-dimensional space.

Additionally, there are other mechanisms to produce the heterogeneity and connectivity that can operate in the viral quasispecies: (i) complementation, which is the supply of a genome (or a fragment of it) that performs a function which can be used by another genome defective in that function^47,48^; (ii) interference, which is the opposite process, namely when a defective virus reduces the biological efficacy of other viral genomes in the same population^9^; and recombination (which acquires its main biological relevance in long-term evolution, once a virus has diversified into multiple lineages) may also play a role within mutant spectra, and therefore this mechanism has also been incorporated into quasispecies theory^49–51^. In the case of RNA viruses with clinical relevance, interaction mechanisms and recombination can have an impact on viral pathogenesis^52^ and on the development of antiviral drug resistance, which is a major cause of antiviral treatment failure^53,54^.

The rhizomatic view of virus quasispecies cannot be reduced to a single type of connection, as a given viral genomic region may engage in different connections. That is, the heterogeneity of the population in that genomic region does not evolve in relation to just one challenge, but to multiple challenges. An example of this is the 5’untranslated region (5’UTR) of hepatitis C virus (HCV) genomic RNA which, besides binding efficiently to the 40S ribosome subunit^55^ and initiating cap-independent translation of the viral genome^56^, also protects the viral RNA against degradation by cellular 5’ exonucleases^57,58^.

In D&G rhizome, heterogeneous elements with very diverse forms of codification are connected. In addition to merely physical or chemical interactions, other different links (syntax, codes, signals) of the rhizome, although incommensurable (in the sense of not being reducible to each other), are not incompatible: there is connectivity and synergy. In a rhizome, the idea of heterogeneity refers both to the incommensurability and to the compatibility of its parts or phases.

The heterogeneity in the nature of the components and links involved in viral signaling processes includes proteins and RNA governed by different syntactic and coding rules that are dependent on the context of complex molecular societies^13,59,60^.

To begin with, the viral genomic regions that do not code for proteins (e.g., HCV 5’UTR) generally follow a repetitive syntax, unlike protein-coding regions, which are not repetitive^59^. In these noncoding regions, the information may be based either on RNA structures (analog), for example internal ribosome entry sites (IRES)^61^, or on the sequence of bases (digital), for example the binding of cellular microRNAs^62^. The RNA sequences and structures of noncoding regions in the genome may help different (coding or noncoding) regions of the viral genome to communicate with each other^63,64^. Alternatively, they may interact with cellular microRNAs^62,65^ or with other highly structured cellular RNAs, such as ribosomal RNA (rRNA)^66,67^. Finally, non-coding RNAs may also interact with viral proteins^68^ or cellular proteins^69^. The other means of coding viral information is via the genetic code that produces viral proteins, which in turn form an extensive network of interactions with the macromolecules in the host cell. It is important to highlight that viral proteins can also interact with both viral RNA and cellular RNA, therefore we should envisage a tangled web of heterogeneous communication channels between viral quasispecies and the cell (a much more complex process than a standard signal transduction) that increases the possibilities of triggering novel interactions.

At this point, we have to consider that communication is a complex activity in constant evolution rather than merely a process of information exchange. There is no neutral and objective information; on the contrary, the meaning of a communicative fact is determined by its context^70^. In addition, and unlike a mechanistic process whose language can be optimized and adjusted for a specific set of elements (e.g., that involved in the regulation of a bacterial metabolic operon, where an inducing molecule is recognized by an operator), there is no ideal “virus/host-cell pair” in the context of a quasispecies. Establishment of paired interactions is subject to the blind experimentation of nature, even under seemingly constant environmental conditions. Typically, a given viral quasispecies can infect a variable range of host cells. For example, evidence of compartmentation of HCV variants has been observed in human brain and lymph node samples, with indications of both phylogenetic and phenotypic clustering^71^. This ability of viral quasispecies to infect new cell types underlies their capacity to jump to new host species^24^. There are numerous examples of viral emergences (including epidemics and pandemics) in nature, such as bat- and rodent-borne RNA viruses that have been cross-transferred to other animal species, including humans^72^, as well as the deeply investigated transmission of the simian immunodeficiency virus to humans, which gave rise to the AIDS pandemic produced by human immunodeficiency virus (HIV)^73^.

### In terms of 3. Principle of multiplicity

**Figure 4:**
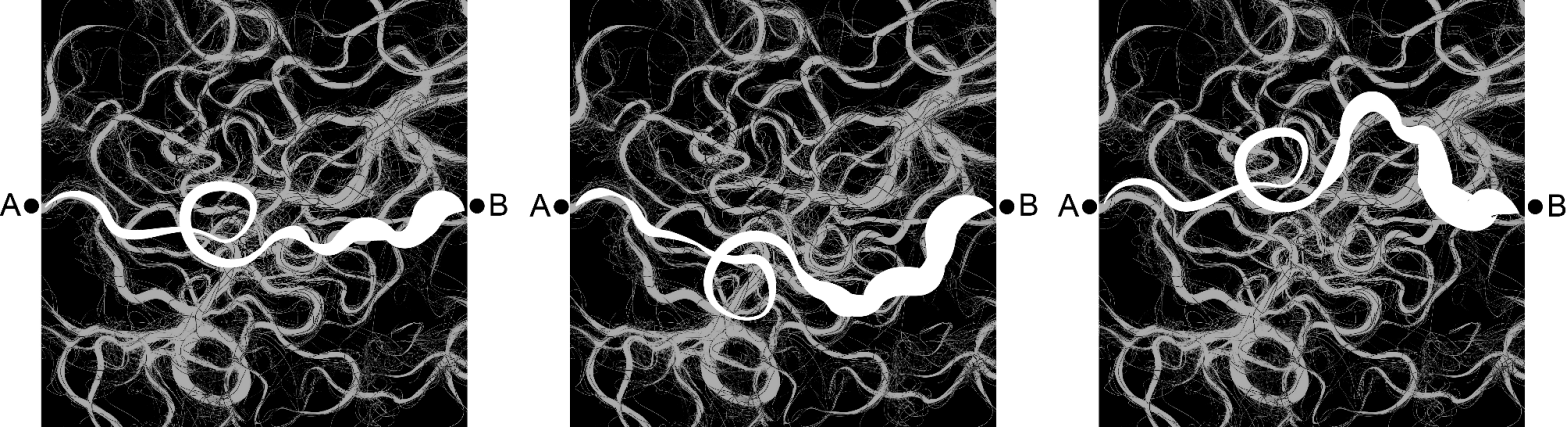
Artistic rhizomatic evolutionary map. Representation illustrating the multiple potential evolutionary trajectories between different positions in a complex sequence space for an evolving viral quasispecies depicted by faint gray lines. Three potential pathways between specific points A and B are marked with thick white lines.

Genetic heterogeneity is not an exclusive property of RNA viruses, as single-cell analyses are increasingly documenting cell-to-cell variations even within the same animal or plant tissue. Host cells provide a plethora of potential, non-identical interactions between the virus and cellular factors. Indeed, many established cell lines used in the experimental evolution of viruses are highly heterogeneous as a result of the genetic instability and epigenetic modifications inherent to replicating tumor cells. These heterogeneities have to be added to environmental variations in their culture medium, such as pH changes and the accumulation of metabolites in the supernatant, amongst others^74^. Heterogeneity is probably more pronounced in natural tissues containing a mixture of cells of different ages, degrees of differentiation, perfusion, etc. As such, there are multiple potential interactions between the heterogeneous viral population and the heterogeneous cell elements, and therefore there is also a multitude of ways by which the virus may adapt to the cell or promote unexpected interactions (Fig. 4).

The transition from a cytolytic infection (involving cell killing) into a persistent infection (coexistence of replicating cells and virus with limited cell killing), which could, a priori, be viewed as an adaptive process towards some cell-virus equilibrium, may result in unexpected outcomes. One such outcome was observed, for example, with the animal pathogen foot-and-mouth disease virus (FMDV) in infections of BHK-21 cells in culture, where persistence was established with a few cells that survived the virus-mediated killing. In the course of persistence, there was a coevolution of cells and of the resident virus, such that the virus became more virulent for the cells, and the cells became progressively more resistant to the virus^75^. These early findings illustrate that viral persistence arises due to interactions between multiple viral and cellular determinants, as further documented by transcriptomic and proteomic results^76^. This is just one of many examples documenting that cells can mobilize alternative resources to cope with an infection.

Ultra-deep sequencing (UDS) techniques allow the simultaneous analysis of viral and cellular RNA, thereby unveiling viral heterogeneity at a single-cell level. For instance, in HCV replicon-infected cells, researchers assessed quasispecies variations across various cell types^45^. Influenza-infected cells also exhibit broad cell-to-cell variation in viral RNA replication^77^, while different amounts of FMDV progeny production in individual cells was documented by single-cell monitoring of infection of a heterogeneous BHK cell population^78^. Host antiviral response heterogeneity is depicted by single-cell sequencing of T- and B-cell receptors (TCR and BCR)^79^, or by examination of individual cells during interpretation of the pathogen-specific immune response^80^.

Indeed, serial passages of HCV in cell culture showed that viral quasispecies evolved despite the cells used for infection being renewed from the same stock in order to avoid cell co-evolution with the virus. Despite this, serial infections did not result in a narrowing of the mutant spectrum. Rather, they resulted in continuous diversification and exploration of new regions of sequence space as the virus gained fitness^81^.

In infections of multicellular organisms, heterogeneity is further accentuated by the viral mutant swarm meeting different cell types in different cell-cycle phases and in different microenvironments. Also, the likely influence of the microbiota on these dynamic virus-host cell interactions should be investigated.

### In terms of 4. Principle of asignifying rupture

This principle relies on the continuous formation and disruption of connections between various variant replicons in a spontaneous manner. In accordance with this principle, in the face of any drastic change affecting the conditions under which the virus quasispecies multiplies, thereby endangering its continuity, the quasispecies can reinitiate on its former or novel trajectories. The application of this principle to RNA viruses is consistent with the enormous difficulty of interrupting or “curing” a viral infection using drugs that inhibit one of the functions coded by the viral genome, because the virus develops mechanisms of antiviral resistance (or, analogously, vaccine resistance)^54^. It has been proposed that viruses have to overcome two barriers to become resistant to an antiviral drug: genotypic and phenotypic^82^. The genotypic barrier concerns the number and type of mutations (transitions or transversions, as well as insertions or deletions) a viral genome must acquire to evade an antiviral agent or a combination of agents. When just one mutation can confer resistance, the (low) genetic barrier can be easily surmounted, given the high mutation rate of RNA viruses^83^. in turn, when multiple mutations are required for resistance, the (high) genetic barrier is more difficult to overcome, because the frequency of occurrence of a genome with the needed constellation of mutations is low, and below the range of population size within an infected individual. This is the basis for the success of combination therapies for RNA viral diseases, including HIV and HCV infections.

The phenotypic barrier considers the fitness cost inflicted upon the virus as a consequence of the acquisition of resistance mutations. This cost is variable and unpredictable, and it may be transient, as the quasispecies may subsequently acquire other compensatory mutations with a positive impact on fitness, partially or totally restoring its original level^82,84^. Another mechanism of viral resistance to multiple drugs that does not rely on specific resistance mutations has been observed with combination treatments administered to patients chronically infected with HCV^85^. In them, highly repeated substitutions generally found at high frequency in the mutant spectrum of HCV are suspected to contribute to (or be markers of) high replicative fitness, which is a critical parameter that can mediate antiviral resistance^82,86,87^. The multiple mechanisms of drug resistance in viruses fit the rhizomatic view we are proposing for viral quasispecies, since they mirror the experimental evidence for multiple, alternative mutational pathways that are available to a virus to gain fitness (^82^, among other examples). Multiple pathways for fitness gain (either following bottleneck events, or to confront an environmental change such as the presence of an antiviral inhibitor) can be equated with multiple lines that relate two rhizomatic nodes, one node being low fitness and the other being high fitness (Fig. 4).

### In terms of 5. and 6. Principles of cartography and decalcomania

With regard to representation, virology (as is the case for other fields of biology) often compares viral sequences and protein structures in different viral genera and families and then visualizes their relationships in the form of phylogenetic trees. It is assumed that the viral diversity is equal to, or greater than, that of the potential host cell^88^. Such trees can be used to draw inferences about a virus’ family relationships, background or history, and often about its origins^89^. This way of working with genetic information is also helpful in the analysis of RNA virus quasispecies, for example when it allowed elucidating the origin of HCV infection in several heart-surgery patients infected by the operating surgeon^90^. However, this approach has a limited value because it only provides a snapshot in time of a subset (collected in the sequenced sample) taken from a much larger and ever-changing set of genetic variants.

Another feature of viral quasispecies is their ability to retain information that was useful in the past in the form of sequence subpopulations within the mutant spectra. This fact represents the replicative memory of a viral quasispecies, initially discovered in FMDV^91^. The quasispecies memory response to a recurring environmental change does not translate into a branch in the population: it is an “advancement” process in which it becomes predominant over the population dominating prior to the challenge, as it was also documented in HIV^92^. This represents a backward movement or a negative change in evolutionary progression.

Quasispecies do not, therefore, adhere to the tree-like organization as their structure changes in the sequence space depending on the cell environment and organism’s response, which are also constantly being transformed. In RNA viruses, it is more useful and more commonplace to apply a set of measurements that capture different aspects of the quasispecies diversity (diversity indices)^93^: some of them provide information on the mutated positions and others about possible selective or disruptive forces (i.e., increase of the mutational input) acting on the population^94^.

Thus, instead of a monotonous bifurcation of the sequence populations that repeat the “tracing” of the evolutionary process as a tree again and again, what we observe are evolutionary lines moving in different directions with multiple intersections and varying rates of evolution. These intersections often merge multiple originally divergent subpopulations, thus resulting in evolutionary lineages that are reinforced or weakened (i.e. with the appearance of non-replicative viruses that block the replication of competent ones). This emphasizes the difficulty of trying to find a single root to the evolutionary phenomenon in RNA viruses.

## A relational definition of viral quasispecies

This definition is grounded in an examination of D&G’s concepts and their relevance to viral quasispecies, which are no longer conceived as a substantial entity or “subject” with various effects on the cell, nor as alien “objects” against which the infected cell (or body) has to defend itself. Instead, they are a configuration of changing interconnections, both internal and external, between the population of viral sequences that are promoted by the high mutation rate of RNA viruses. These interconnections interact, coexist, memorize, combine, and compete among themselves, thereby increasing virus and cell heterogeneity, which subsequently produces more productive interconnections. A rhizomatic viral quasispecies is also the network that forms between heterogeneous viral and cellular elements.

The rhizome defines viral quasispecies as a process in relation to the host cell and provides the logic that can explain the transverse and heterogeneous connections among viruses, as well as of viruses with cells, organisms, and ecosystems. This enables the joint treatment of evolutionary pathways propelled by diverse and sometimes simultaneous selective forces. In addition, despite the irreducibility of these elements, the rhizome unifies the components through their essential commonalities within a specific type of virus.

## Discussion

Herein we have illustrated how the experimental results from studies into the evolution of RNA virus populations agree with D&G’s six principles on the rhizomatic nature of reality. These parallels between viruses and the different rhizome categories give rise to a novel, relational definition of viral quasispecies. This definition complements the widely accepted definition of quasispecies in virology by transitioning from entities to relationships, or from “substance” to “process”^95^. This viewpoint highlights the well-established and high degree of connectivity between points within the RNA virus sequence space, as well as the multiple communication routes facilitated by viral expressed RNAs and proteins. These interactions occur both within genomes of the same viral ensemble as well as between various components of host cells that support viral replication, thus involving organisms, their immune response and even their ecosystems. The rhizome concept therefore becomes a framework for describing the heterogeneous space of viral variant relationships, encompassing physicochemical influences as well as sign-mediated context-dependent interactions^96^.

Our initial characterization suggests that we can apply the rhizomatic concept to interpret data and previous discussions of quasispecies in a fundamental tenet that diverges from (and enriches) traditional virology. Thus, virology attributes changes in infected cells or organisms to a specific agent (a “substance”) that forms the focal point of research from a philosophical perspective. This represents the “molar” approach to viral genetic information, as defined by the consensus sequence that portrays the virus as the ultimate cause of infection. However, experiments in RNA virus quasispecies have reconfigured this view by demonstrating that there is no such thing as “one mol” of an RNA virus (as a substance defined by a consensus sequence), as it is really a collection of variant sequences^9,90,91^ and each particular variant has only a fleeting or statistical existence.

In viral evolution, classic “arboreal” thinking operates on the premise that viral divergence leads to a hierarchical branching pattern, from one consensus sequence (type A) to two different consensus sequences (types B and C). Therefore, this model prioritizes identity over differences^97^ and neglects interconnections between branches within the evolutionary flow. Conversely, rhizomatic organization highlights a decentralized and interconnected network, considering pathways of descent beyond the traditional linear progression of a phylogenetic tree^22^. Quasispecies experiments have focused specifically on the singularities of RNA virus evolution, virus–host communication, and resistance to drugs and vaccines evasion, and thus have prioritized discovering differences over identity^98^.

The ability of the rhizome to incorporate different types of physicochemical and sign-mediated relationships into its modular extensions allows us to integrate apparently unrelated virus behavior and virus–cell descriptions and entities at different biological levels. In particular, the rhizome allows us to consider participation in the evolutionary process of both each variant sequence and the whole population, by placing an emphasis on the flow between the two through a dynamic stabilization and de-structuring of the population. For example, the individual variant alliances, namely progeny sharing via a high mutation rate, complementation, recombination, and virus–cell co-evolution, tend to establish a population organization, whereas continuous competition between different sub-groups, defective interfering viruses, and transmission bottlenecks leads to population fragmentation^98,99^. This dynamics contrasts with approximating the individual variant or “the mol of virus” as different entities with distinct biological properties. The rhizome concept does not oppose the population and the individual. Instead, both entities are considered integral to the evolutionary process. For instance, regarding viral robustness in response to environmental change, the explanation relies on the proximity relationship of variant sequences in the sequence space among the members of the population, thus meaning that the whole quasispecies is responsible for this kind of response^100,101^. Conversely, during the colonization of a new host, individual variants (free from the population restrictions for a few replication rounds) take on the responsibility for the fate of a new infection.

In the rhizome framework, the interconnectivity and inter-influence of viral variants acquire a new type of visibility^102^, and the trajectories linking any two points in the network (mainly via mutation and recombination pathways), and their thicknesses (depicted in Fig. 4), vary over time. These variations arise due to the random occurrence of genetic alterations and chance encounters between viral mutants or with the heterogeneous repertoire of host cell molecules. In extreme cases, they may even arise due to changes in host species that cause unpredictable changes in fitness. The thickness of a trajectory is given by the relative fitness of the set of related genomes placed in that trajectory (see the thick and thin branches in Fig. 4). Fitness can be quantified experimentally by the frequency of the different haplotypes in the mutation spectrum, as determined by UDS techniques performed at any point in an infectious process. The highly dynamic variation in trajectories (not just in their thickness) fits with the observations reviewed in previous sections of this work describing multiple, alternative pathways for traveling between two points of the rhizome. Our current understanding is that a deviation towards a different trajectory (start of a new branch) often begins as a random event, as is the nature of mutation and recombination, due to quantum–mechanical uncertainties in base-pairing involved in nucleotide recognition. The influence of random events mirrors an increasing trend towards “stochastic thinking” in the biological sciences^103^.

Stochasticity can explain features of both viruses and cellular collectivities, as well as some critical events in the enzymology of DNA or RNA replication. The fact that enzymes, viruses, and cells receive a critical contribution from stochastic events (instead of only regulated gene expression processes and metabolic reactions) reinforces the general rhizomatic view of life. Beyond the initial random mutation, the possibility of multiple new trajectories arises due to the diverse heterogeneities within the cell, organism, or ecosystem, whether latent or patent, that can be activated or exploited during viral infection^13^. Hence, a novel interface of stochasticity involving the fortuitous convergence of a new mutation with an alternative heterogeneity, becomes imperative to establish a new trajectory. Once new trajectories have been triggered by random mutations and encounters, environmental demands activate competitive and selective responses, thus producing more branches and thickness variations.

Taking into account the ability of viral quasispecies to respond to, adapt to, integrate and transfer different forms of biological variability, we could argue that RNA viruses contribute to the formation of a rhizome of biological variability in nature. In this regard, a critical event in viral populations that accentuates their participation in the rhizomatic network of life is the modification of host cell tropism as a result of mutation or recombination. Barriers that prevent the infection of new cell types in a given host (or, less frequently, cells of different species) are more difficult to estimate than those that have to be surmounted to acquire resistance to antiviral agents. Despite this, examination of different cases of cell tropism modification by viruses suggests that minimal numbers of genomic changes may suffice for a change in host cell tropism. This has multiple implications for both viral disease emergence and the promotion of HGTs between cells^94^, and is a means for viral quasispecies to create different degrees of connectivity in the sense of D&G.

The rhizome concept shares several key points with biocommunicative theory as both focus on organization and coordination^14^. Moreover, both reject a universal grammar or a fixed structure (whether Porfirian three, Linnaean taxonomies or Chomsky diagrams) linking different elements^14^. In the rhizome these connections are made through their sensing and response to changes, and are thus established pragmatically according to need: they are the result of experimentation. Furthermore, in biocommunication, sign-mediated rules for each suborganization (rhizome protrusion here) would be shaped by their own evolutionary history, which is compatible with the rhizomatic view, and these remain open to unpredictable incorporations, ruptures, and lines of flight, which is difficult to reconcile with the traditional concept of biological systems.

The rhizome is “an image of thought”^97^, as described by D&G, and provides a space for knowledge classification that is lacking in the proposals of other authors who have moved away from classical Aristotelian categories, such as Ludwig Wittgenstein and John L. Austin, and thus represents a offers a useful complement to classify the results of biocommunicative studies^104^. Another viable image would be that of an encyclopedia proposed by Humberto Eco^102^. The rhizome metaphor encourages us to explore connections where we might usually presume there are none^105^.

Current explorations or approaches in virology based on lethal mutagenesis, which tend to increase the mutation rate of a pathogenic virus as a way to eliminate it, can be interpreted as solvents of the viral population structure by directly weakening the connections between the variants in the population, and would align with the rhizomatic vision of viral quasispecies.

The role of stochasticity’s permanent participation in modulation of the rhizomatic bush is still open for interpretation. Little is known of the time frame in which all the events modulating rhizomatic conformations occur, and this is one of the many challenges for future study. Also, the rhizomatic approach brings us closer to tackling the paradox that evolution mainly relies on the changes induced by viruses (such as enabling internal gestation in mammals) even as we, as cellular organisms, must defend ourselves against them^106^. This perspective also implies that viruses might constitute an intrinsic component of an organism’s biology rather than being regarded as foreign entities^106,107^. Future work should shed further light on the explanatory power of conceptualizing RNA quasispecies in a rhizomatic manner.

## Conclusions

Viral quasispecies and their role in nature may be thought of, and defined, in terms of the rhizome as this accounts for the unstable and dynamic nature of the composition of the viral population, which is always in transit within virus/host communication. The rhizomatic view of RNA virus populations helps the molar/molecular (consensus sequence/individual variant) dichotomy to be overcome.

It also offers a means of understanding the channeling of random events, mainly due to nucleic acid mutations in viral genomes, but also encounters and ruptures bridging the gap between the virtual and the real.

The activity of viral variants is dependent on their communication with themselves and with the intrinsically heterogeneous cells for countless repeated cycles of infection. Thus, variability in virus-host ensemble becomes connected, blurring the frontiers between virus and cells/organism evolution, as well as the differences between subject and object.

Rhizomatic quasispecies elements possess evolutionary history and are open to unpredictable incorporations and ruptures. In the face of an epidemic or pandemic, for instance, this therefore suggests the need to establish a multifaceted community defense against viral transmission instead of relying solely on vaccination^108^, which treats the virus as an inert toxic entity, an object or “a solid” in terms of Henri Bergson ^109^:

(…) the human intellect feels at home so long as we allow it to remain among inert objects, particularly among solids, where our action finds its footing and where our industriousness finds its tools. We will see that our concepts have been formed in the image of solids and that our logic is, above all else, the logic of solids. And we will see that, for the same reasons, our intellect excels in geometry, where the kinship between logical thought and inert matter is revealed and where the intellect, after the lightest possible contact with experience, need only follow its natural movement to go from one discovery to the nest with the certainty that experience marches along behind it and will invariably prove it right.

But it also follows from this that our thought, in its purely logical form, is incapable of conceiving of the true nature of life and the deep meaning of the evolutionary movement. (*Creative Evolution)*

**Box 1**: The origins of the debate lie in pre-Socratic philosophy, in ideas developed by Heraclitus of Ephesus and Parmenides of Elea. In contrast to the notion of “what prevails”, Heraclitus placed an emphasis on the importance of constant change and flux, as he conceived the world to be in a state of *continuous becoming* balanced by the *conflict of opposites*. Unlike the theory of a changing, transitory, and fluctuating world, Parmenides believed in the importance of “what prevails”, of what is stable and eternal, as the ultimate foundation of reality ^110^.

Historically, the hegemonic “common sense” of Western philosophical and scientific thinking prioritized Parmenides’ theory, and thus the paradigm of substance metaphysics. His school of thought has been developed in various guises throughout history: Leucippus’ and Democritus’ atomism, Platonic ideas, Aristotle’s substances, modern atomic theory, and so on. With the exceptions of some schools, until the advent of quantum mechanics and the wave-particle duality in the twentieth century, scientific development was founded on the principle that scientific theory must revolve around the characteristics of things that we perceive or suppose as essentially fixed and stable in their essence.

Heraclitus’ view was redeemed in various forms through the philosophy of different thinkers, such as Hegel, Nietzsche, Whitehead, Bergson, Deleuze, Guattari, and, contemporaneously, Dupre.

**Box 2:** A river, shaped by its surroundings, constantly changes as it flows through diverse landscapes. Its essence lies in the dynamic interaction between water, rocks, and vegetation, influenced by various factors like terrain, rainfall and temperature. This dynamic relationship gives rise to its intensive-virtual potential, while its observable form in the real world reflects its extensive-actual manifestation. Ultimately, the river embodies a complex “multiplicity” shaped by countless elements and factors.

**Box 3:** In a game of chess, players envision future moves, each representing a virtual combination within the game's rules and current position. These potentialities shape the game's outcome, creating a dialectic between intensive-virtual possibilities and extensive-actual moves on the board.

## Data Availability

All data generated or analyzed during this study are included in this published article.
